# Use and improvement of microbial redox enzymes for environmental purposes

**DOI:** 10.1186/1475-2859-3-10

**Published:** 2004-08-02

**Authors:** Humberto Garcia-Arellano, Miguel Alcalde, Antonio Ballesteros

**Affiliations:** 1 Departamento de Biocatálisis, Instituto de Catálisis, CSIC, Cantoblanco, 28049 Madrid, Spain

## Abstract

Industrial development may result in the increase of environmental risks. The enzymatic transformation of polluting compounds to less toxic or even innocuous products is an alternative to their complete removal. In this regard, a number of different redox enzymes are able to transform a wide variety of toxic pollutants, such as polynuclear aromatic hydrocarbons, phenols, azo dyes, heavy metals, etc. Here, novel information on chromate reductases, enzymes that carry out the reduction of highly toxic Cr(VI) to the less toxic insoluble Cr(III), is discussed. In addition, the properties and application of bacterial and eukaryotic proteins (lignin-modifying enzymes, peroxidases and cytochromes) useful in environmental enzymology is also discussed.

## Introduction

Chromate reductases are a group of enzymes that catalyze the reduction of toxic and carcinogenic Cr(VI) to the less soluble and less toxic Cr(III). These proteins have recently raised enormous interest because of their central role in mediating chromium toxicity and their potential use in bioremediation and biocatalysis. Chromate (Cr(VI)) is generated as by-product of various industrial processes such as leather tanning, chrome-plating, pigment production and thermonuclear weapon manufacture [1]. Its high water solubility facilitates a rapid leaching, provoking a wide dispersion capable to contaminate drinking water supplies. Therefore, the characterization of enzymes that reduce chromate, as well as the study of their induction patterns and gene expression are relevant to complete our understanding of chromium metabolism in order to minimize the toxicity of this compound in the environment.

The chromate-reducing activities have been located in the cell membrane or in the cytoplasm of many bacteria [2]. Their ubiquities in many different organisms suggest that they might share a common role in, for example, physiological redox sensing or detoxification. Recently, two novel dimeric flavoproteins with chromate reductase activity, ChrR (from *Pseudomonas putida*) and YieF (from *E. coli*) have been purified and characterized [1]. These enzymes were able to transform chromate to the less toxic Cr(III). However, while ChrR was not a pure two-electron reducer of chromate, YieF was able to catalyze a three-electron reduction. The role of ChrR and YieF in protection against chromate toxicity was also investigated and the results suggested that both enzymes may have an important role in protection against chromate toxicity [1].

The ability of some microorganism and their enzymes to remove toxic pollutants has been recently reviewed [3-6]. The identification and characterization of the degradative pathways functioning in microorganism have been the starting point for biotechnological and environmental applications [3].

## Discussion

The intensive industrial and agricultural development has been considered as responsible for a widespread contamination of soil, air and groundwater with toxic pollutants, which are harmful for human health and the environment [6]. These contaminants enter the environment through different paths, which may include direct application, combustion processes and natural emissions. Major contaminants are polycyclic aromatic hydrocarbons (PAHs), petroleum hydrocarbons, phenols, polychlorinated biphenyls, azo dyes, organophosphorus pesticides and heavy metals [3]. In particular, Cr(VI) is a common pollutant due to the use of chromium compounds in tanning and other industries. Chromate shares structural similarities with sulphate ion (SO_4 _^-2^) and may be introduced in eukaryotic and bacterial cells by the sulphate transport system [1]. In bacteria, flavoenzymes such as glutathione reductase reduce Cr(VI) by a one electron transfer leading to the formation of the highly unstable radical Cr(V) and the flavin semiquinone form of the enzyme. Both species undergo a further redox cycle in which Cr(VI) is re-generated by one-electron transfer to oxygen, producing and accumulating reactive oxygen species (ROS). The appearance of relatively large quantities of ROS, and the consequent oxidative stress are responsible for the toxic effects and cellular damage attributable to the presence of Cr(VI). On the other hand, trivalent chromium Cr(III) is water insoluble, less bio-available and less toxic [1]. Thus, the strategies employed to eliminate chromate toxicity would involve its reduction to Cr(III) by chemical or biological means.

While chemical methods are expensive at the large scale required to decontaminate waste sites, microorganisms are commonly used for environmental purposes through the exploitation of their natural catalytic activities. Enzymatic treatments have a minimal impact on ecosystems, as they present no risk of biological contamination. Furthermore, enzymes can act over a wide range of pH, temperature and ionic strength and also may be active in the presence of high concentrations of organic solvents in which major pollutant molecules are soluble [6].

Several bacterial enzymes that can be used in bioremediation have been described; they include mainly oxidative enzymes such as mono-and dioxygenases but their use is restricted by the need of cofactors, which can only be efficiently regenerated inside the microorganism [6]. In the last two decades bioremediation has explored the use of the catalytic machinery of white rot fungi to remove toxic pollutants. White rot fungi comprise all those fungi capable to degrade lignin, a polyphenolic polymer highly resistant to bacterial biodegradation. Many strains from the genera *Pleurotus, Bjerkandera, Phanerochaete*, and *Trametes *produce extracellular enzymes with ligninolytic activity. These enzymes are often referred to as lignin-modifying enzymes and include mainly Lignin peroxidase, Manganese dependent peroxidase and laccase [4], though some authors have reported other related enzymes such as a Mn-independent MnP activity [7]. Besides lignin-modifying enzymes, several other enzymes such as the heme-containing peroxidases, chloroperoxidase and horseradish peroxidase, and the non-enzymatic hemeproteins, hemoglobin and cytochrome *c*, are able to oxidize organic compounds in the presence of hydrogen peroxide. An interesting feature of these enzymes is their remarkable low specificity towards substrates that arises from their own catalytic mechanism. *In vivo*, peroxidases use endogenous low-molecular weight compounds, called mediators, to generate free radicals capable to carry out a wide variety of reactions such as oxidations, bond cleavage, hydroxylations, polymerization and demethylation [4]. Several research efforts have been focused on the ability of peroxidases to degrade pollutants such as PAH's, azo dyes and organophosphorus pesticides [8-10].

Strong regulations have been established to push the industrial sector to develop new programs destined to a greater environmental care. Nowadays, industry is strongly dependent on petroleum and its derivatives as a source for raw materials and energy. There are still large reserves of crude oil, which are heavy oils with a high content of sulphur and heavy metals. The use of these fuels generate a great pollution, being one of their most important environmental impacts the formation of the acid rain which takes place by the sulphur oxide production during combustion. Redox enzymes may encounter fields of application not only in the bioremediation of polluted environments, but also in the development of novel clean technologies to avoid or diminish the environmental contamination. Biocatalytic methods for sulphur removal from straight-run diesel fuel have been developed [11]. The removal of heavy metals from the petrophorphyrin-rich fraction of asphaltenes has also been reported [12,13]. Thus, enzymes can play an important role in the development of alternative or complementary biotechnological processes with potential application in polluting industries.

Despite their potential application in bioremediation and clean processes, the activity of oxidative enzymes may be limited, among other factors, by the low bioavailability of the pollutants and by the relatively low operational stability of the enzyme under the environmental conditions required to carry out the bioremediation. Several strategies to increase the catalytic activity of peroxidases have been proposed, including chemical modification of the enzyme [14,15] and genetic tools [16]. Recently, with the cloning and expression in suitable hosts, larger amounts of the desired enzymes may be produced, facilitating their characterization and their direct use in environmental applications. Further, through the use of novel techniques such as directed molecular evolution [17], proteins designed specifically for bioremediation could be made available in a not distant future. In the last few years there has been an extensive research in the application of laboratory evolution for tailoring redox enzymatic systems (laccases, peroxidases, cytochrome P450 monooxygenases) to improve their activities and stabilities against temperature or organic solvents [18-20]. The application of this powerful approach for bioremediation issues is coming up, but first a big effort in the high-throughput (HTP) screening methodology must be done. So far, little has been reported on the optimisation of suitable HTP for the detection of xenobiotics [21]. Therefore, the success in the enzyme evolution for environmental issues will be highly dependent on the automation of HTP.

Microbial genomics is a new emerging field that enables us to look at parts of the environment that were, until recently, masked to us. Present estimations suggest that more than 99% of the microorganisms in most environments (also those subjected to chronic contamination) are not amenable to grow in pure culture, and thus very little is known about their enzymatic activities. We can now access the genomes of non-culturable microorganisms through creating the "so-called" metagenomic-libraries and identify protein-coding genes and biochemical pathways that will shed some light on their properties and function [22]. New enzymatic systems found in contaminated areas can be used as parental types for some rounds of directed evolution with the main aim of improving the catalytic performance for their use towards solving a broad range of environmental problems.

## Conclusion

The even more strict regulations on hazard wastes has forced to the development of new environmentally compatible strategies to substitute or complement the conventional ones. Chemical technologies are expensive when applied to large scale and in many cases are technically not feasible. On the other side, by exploitation of the huge diversity of natural activities and metabolic pathways presented by microorganisms, new strategies can be envisaged. The use of oxidative enzymes as biocatalysts for environmental purposes presents a promising potential due to their low specificity and low energetic requirements. However, further characterization of new biocatalysts is needed. The use of novel technologies such as molecular directed evolution may have a large impact in the tailoring and further application of enzymes not only for bioremediation but also for the development of friendly environmental technologies.

## References

[B1] Ackerley DF, Gonzalez CF, Park CH, Blake IIR, Keyhan M, Matin A (2004). Chromate reducing properties of soluble flavoproteins from *Pseudomonas putida *and *Escherichia coli*. Appl Environ Microbiol.

[B2] Kwak YH, Lee DS, Kim HB (2003). *Vibrio harveyi *nitroreductase is also a chromate reductase. Appl Environ Microbiol.

[B3] Dua M, Singh A, Sethunathan N, Johri AK (2002). Biotechnology and bioremediation: successes and limitations. Appl Microbiol Biotechnol.

[B4] Pointing SB (2001). Feasibility of bioremediation by white-rot fungi. Appl Microbiol Biotechnol.

[B5] Pieper DH, Reineke W (2000). Engineering bacteria for bioremediation. Curr Opin Biotechnol.

[B6] Torres E, Bustos-Jaimes I, Le Borgne S (2003). Potential use of oxidative enzymes for the detoxification of organic pollutants. Appl Catal B-Environ.

[B7] Maximo C, Amorim MTP, Costa-Ferreira M (2003). Biotransformation of industrial reactive azo dyes by *Geotrichum sp *CCMI 1019. Enzyme Microb Technol.

[B8] Majcherczyk A, Johannes C, Huttermann A (1998). Oxidation of polycyclic aromatic hydrocarbons (PAH) by laccase of *Trametes versicolor*. Enzyme Microb Technol.

[B9] Rodriguez E, Pickard MA, Vazquez-Duhalt R (1999). Industrial dye decolorization by laccases from ligninolytic fungi. Curr Microbiol.

[B10] Hernandez J, Robledo NR, Velasco L, Quintero R, Pickard MA, Vazquez-Duhalt R (1998). Chloroperoxidase-mediated oxidation of organophosphorus pesticides. Pestic Biochem Physiol.

[B11] Ayala M, Tinoco R, Hernandez V, Bremauntz P, Vazquez-Duhalt R (1998). Biocatalytic oxidation of fuel as an alternative to biodesulfurization. Fuel Process Technol.

[B12] Fedorak PM, Semple KM, Vazquez-Duhalt R, Westlake DWS (1993). Chloroperoxidase-mediated modifications of petroporphyrins and asphaltenes. Enzyme Microb Technol.

[B13] García-Arellano H, Buenrostro-Gonzalez E, Vazquez-Duhalt R (2004). Biocatalytic transformation of petroporphyrins by chemical modified cytochrome *c *. Biotechnol Bioeng.

[B14] Vandertol-Vanier HA, Vazquez-Duhalt R, Tinoco R, Pickard MA (2002). Enhanced activity by poly(ethylene glycol) modification of *Coriolopsis gallica *laccase. J Ind Microbiol Biotechnol.

[B15] Tinoco R, Vazquez-Duhalt R (1998). Chemical modification of cytochrome *c *improves their catalytic properties in the oxidation of polycyclic aromatic hydrocarbons. Enzyme Microb Technol.

[B16] Harford-Cross CF, Carmichael AB, Allan FK, England PA, Rouch DA, Wong LL (2000). Protein engineering of cytochrome P450(cam) (CYP101) for the oxidation of polycyclic aromatic hydrocarbons. Protein Eng.

[B17] Arnold FH (2001). Combinatorial and computational challenges for biocatalyst design. Nature.

[B18] Bulter T, Alcalde M, Sieber V, Meinhold P, Schlachtbauer C, Arnold FH (2003). Functional expression of a fungal laccase in *Saccharomyces cerevisiae *by Directed Evolution. Appl Environ Microbiol.

[B19] Salazar O, Cirino PC, Arnold FH (2003). Thermostabilization of a cytochrome P450 peroxygenase. Chembiochem.

[B20] Wong TS, Arnold FH, Schwaneberg U (2004). Laboratory evolution of cytochrome P450 BM-3 monooxygenase for organic cosolvents. Biotechnol Bioeng.

[B21] Alcalde M, Bulter T, Arnold FH (2002). Colorimetric assays for biodegradation of polycyclic aromatic hydrocarbons by fungal laccases. J Biomol Screen.

[B22] Lorenz P, Liebeton K, Niehaus F, Eck J (2002). Screening for novel enzymes for biocatalytic processes: accessing the metagenome as a resource of novel functional sequence space. Curr Opin Biotechnol.

